# Replication of Association of the *PTPRC* Gene With Response to Anti–Tumor Necrosis Factor Therapy in a Large UK Cohort

**DOI:** 10.1002/art.33381

**Published:** 2012-02-28

**Authors:** Darren Plant, Rita Prajapati, Kimme L Hyrich, Ann W Morgan, Anthony G Wilson, John D Isaacs, Anne Barton, the Biologics in Rheumatoid Arthritis Genetics and Genomics Study Syndicate

**Affiliations:** 1 University of ManchesterManchester, UK; 2 University of LeedsLeeds, UK; 3 University of SheffieldSheffield, UK; 4 Newcastle UniversityNewcastle upon Tyne, UK

## Abstract

**Objective:**

Several rheumatoid arthritis (RA) susceptibility variants map close to genes involved in the tumor necrosis factor (TNF) signaling pathway, prompting the investigation of RA susceptibility variants in studies of predictors of response to TNF blockade. Based on a previously reported association of RA with the *PTPRC* genetic locus, the present study was undertaken to test established RA susceptibility variants, including *PTPRC*, in the prediction of response to TNF blockade in a large cohort of patients from the UK.

**Methods:**

DNA was extracted from the blood of 1,115 UK patients with RA who were receiving anti-TNF biologic therapy. Samples were analyzed for 29 single-nucleotide polymorphisms (SNPs) previously established as RA susceptibility variants. In the primary analysis, the effect of each SNP on treatment response was assessed by linear regression, using an additive model, in which absolute change in the Disease Activity Score in 28 joints at 6 months of followup was the outcome measure. In a secondary analysis, logistic regression models were used to compare patients with a good treatment response (n = 274) to those with a poor response (n = 195), as defined using the European League Against Rheumatism response criteria. Results were combined with those from previous studies to confirm the findings by meta-analysis.

**Results:**

The *PTPRC* rs10919563 SNP was associated with improved treatment response in both the primary analysis (regression coefficient 0.19, 95% confidence interval [95% CI] 0.09, 0.37; *P* = 0.04) and secondary analysis (odds ratio 0.62, 95% CI 0.40, 0.95; *P* = 0.03). A meta-analysis combining these data with the results from a previous study strengthened the evidence for association with the *PTPRC* SNP (*P* = 5.13 × 10^−5^)*.* No convincing association of the treatment response with other candidate loci was detected.

**Conclusion:**

Presence of the rs10919563 RA susceptibility variant at the *PTPRC* gene locus predicts improved response to anti-TNF biologic therapy. Fine-mapping studies are required to determine whether this SNP or another variant at the locus provides the greatest predictive accuracy for treatment response.

The introduction of anti–tumor necrosis factor (anti-TNF) biologic drugs in the clinical management of rheumatoid arthritis (RA) has proven highly successful in suppressing both inflammation and joint damage in many of the treated patients (1). However, although largely effective, biologic drugs are expensive (∼$15,000 per patient per year) and are a potential source of serious toxicity (2). Moreover, up to one-third of the patients are nonresponsive to treatment (1, 3). Ideally, physicians would like to identify which patients are likely to respond to TNF blockade early in clinical management, and both clinical and demographic factors are known predictors of the treatment response (2). Concurrent methotrexate therapy, functional disability, smoking habits, and sex are known prognostic factors for prediction of the treatment response, but these factors account for only a modest proportion of the variance observed (2, 4). The identification of additional, nonclinical factors, which would refine the accuracy of predicting the anti-TNF treatment response, would be a huge clinical advance. Genetic markers may offer one such source.

Most studies of genetic predictors of the anti-TNF response have, to date, focused on candidate genes (5), with only two unbiased studies of the entire genome having been conducted (6, 7). Much of the focus of these studies has been on the *TNF* gene itself (8–10), or on candidate genes in the TNF and related signaling pathways (11–13), as well as on various cytokines (14). However, no single gene influencing the anti-TNF response in RA has been definitively identified and replicated, although evidence indicating a role for the TNF-308 polymorphism in RA remains compelling (4, 15). Likely explanations for this limited success are 1) the small sample sizes utilized by many of the studies, and 2) the predominant focus on candidate genes that have a low prior probability of being associated.

Recently, a number of established RA genetic susceptibility loci, found to be associated with RA susceptibility at a low-to-moderate level of risk, have been identified through genome-wide association (GWA) studies and related meta-analyses (16–21). Interestingly, a number of the identified susceptibility markers map proximally to genes encoding proteins involved in TNF signaling, including the *REL*, *TNFAIP3*, *TRAF6*, and *PTPRC* genes (16, 20, 21). These markers represent attractive candidate loci for the investigations of response to TNF antagonists, since prior evidence has indicated a role of these markers in disease development and proximity to genes that have recognized roles in TNF signaling (22).

Previous investigations of RA susceptibility markers in determining the response to anti-TNF drugs have found that neither the *HLA–DRB1* shared epitope nor the *PTPN22* locus is correlated with the response to biologics treatment (23). In contrast, a putative association between a single-nucleotide polymorphism (SNP) at the *AFF3* locus (24) and anti-TNF response has been observed, but is yet to be confirmed in independent sample collections.

In 2010, Cui et al reported results from a well-powered investigation, which included 1,283 samples from RA patients of European ancestry and investigated candidate markers that have previously been attributed to risk of RA development (22). The investigation assessed the association of the *PTPRC* locus (rs10919563) with response to TNF blockade, where the minor allele (A) of the *PTPRC* locus was associated with a poor treatment response. Protein tyrosine phosphatase receptor type C was first identified as a susceptibility locus for RA in a meta-analysis performed by Raychaudhuri et al (21). The product of the *PTPRC* gene is known to have a role in TNF signaling, and thus represents an intriguing candidate for further investigation.

The aims of the current study were, first, to validate the reported association of rs10919563 mapping to the *PTPRC* gene locus and, second, to investigate other recently identified RA susceptibility markers as predictors of anti-TNF treatment efficacy in a large cohort of UK patients.

## PATIENTS AND METHODS

### Markers

A panel of 35 established RA susceptibility markers was selected for genotyping, based on the findings from the most recent meta-analysis of GWA studies (16, 17). Susceptibility markers that were identified previously in the same UK population of anti-TNF–treated RA patients were excluded (24).

### Patients

The British Society for Rheumatology Biologics Register was initiated with the aim of assessing the adverse events associated with treatment with 3 anti-TNF biologic drugs, etanercept, infliximab, and adalimumab, and has detailed clinical and response criteria identified in 4,000 patients with RA receiving each drug in the UK. Collaborations with a subset of the larger prescribing centers were established as part of the Biologics in Rheumatoid Arthritis Genetics and Genomics Study Syndicate (BRAGGSS) (see Appendix A). Blood samples for DNA extraction were obtained from anti-TNF–treated patients with RA who met the following inclusion criteria: 1) a diagnosis of RA confirmed by a physician; 2) receiving, or about to begin, treatment with 1 of the 3 anti-TNF drugs (etanercept, infliximab, or adalimumab); and 3) Caucasian ancestry (self-reported). Patients were ineligible for this study if they had stopped treatment during the first 6 months for reasons other than inefficacy.

### Genotyping

DNA samples were genotyped using the Sequenom MassArray iPLEX system. In each reaction, 10 ng of DNA was used and the protocol was followed according to the manufacturer's instructions (http://www.Sequenom.com). For purposes of quality control, a 90% sample threshold and 90% genotyping success threshold were used.

### Statistical analysis

Multivariate linear regression analyses were performed to assess the effect of each SNP genotype on response to treatment, using the absolute change in the Disease Activity Score in 28 joints (DAS28) (25) at 6 months of followup (a continuous variable) as the primary outcome measure. In a secondary analysis, multivariate logistic regression models were investigated to compare patients with a good response to TNF antagonists to those experiencing a poor treatment response, as defined by the European League Against Rheumatism (EULAR) response criteria (26). For patients to be classified as a good responder, they must have shown a significant improvement in the DAS28 (defined as a decrease in the DAS28 of >1.2 units) and have achieved a low level of disease activity (defined as a DAS28 score of ≤2.4) by end point. A classification of poor response was defined as change in the DAS28 of ≤0.6 units and a high level of disease activity (DAS28 score of >5.1) by end point.

All SNP associations were analyzed with reference to the minor allele. Regression analyses were adjusted for covariates that had been identified in the BRAGGSS cohort as independent predictors of change in the DAS28: baseline DAS28, baseline Health Assessment Questionnaire (HAQ) score (27), concurrent disease-modifying antirheumatic drug (DMARD) therapy, and sex. These analyses were performed using Plink statistical software (version 1.07; http://pngu.mgh.harvard.edu/purcell/plink/). No adjustment was made for anti–cyclic citrullinated peptide (anti-CCP) antibody positivity, but a subgroup analysis of patients positive for anti-CCP was performed. No adjustment was made for anti-TNF drug type, as the aim was to identify a class effect. However, in order to establish whether a drug type–specific effect existed at any of the associated loci, an interaction term between SNP loci and drug type (i.e., etanercept, infliximab, or adalimumab) was fitted.

For the marker mapping to the *PTPRC* locus, a meta-analysis that included data from previously published studies (22) was also performed. Eighty-one overlapping samples were removed before conducting the meta-analysis. Fixed-effects models (using the Mantel-Haenszel method) were constructed, and studies were weighted according to the amount of information they contained. Cochran's Q and I^2^ test statistics were used to estimate the variation in effects attributable to heterogeneity, and *P* values were determined by chi-square test. These analyses were performed using Stata statistical software (http://www.stata.com). Power calculations were performed using Quanto (version 1.2.3; http://hydra.usc.edu/gxe) under an additive model, for a range of marker allele frequencies.

## RESULTS

A total of 35 SNP markers previously identified as RA susceptibility variants were genotyped in 1,387 DNA samples from patients receiving TNF blockade therapy. Following genotype and sample quality control, 29 SNP markers remained available for analysis in 1,270 samples (further details available from the corresponding author upon request). Six SNP markers (rs12746613 [1q12], rs934734 [*SPRED2*], rs10488631 [*IRF5*], rs3184504 [*SH2B3*], rs7155603 [*BATF*], and rs11203203 [*UBASH3A*]) failed to meet the imposed quality control threshold. Of 1,270 patients, 155 were ineligible for analysis, for the following reasons: 98 patients stopped treatment with biologics for reasons other than efficacy, 4 patients changed biologic agents, and 53 patients had a missing baseline or 6-month DAS28 value.

The study had >90% power to detect a difference in the DAS28 of ≥0.6 units (a clinically meaningful change) for allele frequencies of >5%. The demographic and clinical characteristics of the 1,115 patients available for analysis are presented in Table 1.

**Table 1:** Baseline clinical and demographic characteristics of the 1,115 patients with rheumatoid arthritis*

Age, mean ± SD years	56.5 ± 11
No. (%) female	855 (77)
No. (%) current smokers	179 (16)
Disease duration, median (IQR) years	12 (6–19)
DAS28 at baseline, mean ± SD	6.66 ± 0.98
HAQ score at baseline, median (IQR)†	2.125 (1.75–2.50)
No. (%) treated with concurrent DMARDs	819 (73)
No. (%) treated with etanercept	416 (37)
No. (%) treated with infliximab	442 (40)
No. (%) treated with adalimumab	257 (23)
No. (%) treated with previous biologic therapy	71 (6)
No. (%) autoantibody positive‡	592 (93)

* IQR = interquartile range; DAS28 = Disease Activity Score in 28 joints; HAQ = Health Assessment Questionnaire; DMARDs = disease-modifying antirheumatic drugs.

† Data available on 1,066 patients.

‡ Data on autoantibody status (positivity for anti–cyclic citrullinated peptide antibodies/rheumatoid factor) available on 639 patients.

In the initial multivariate analysis, which used the absolute change in the DAS28 over 6 months as the primary outcome measure, presence of the major allele G, which is also the RA susceptibility allele, at the *PTPRC* locus (rs10919563) was associated with an improved response to TNF blockade therapy, as determined under an additive model (regression coefficient 0.19, 95% confidence interval [95% CI] 0.09, 0.37 versus carriers of allele A; *P* = 0.04) (Table 2). This association was corroborated in the secondary analysis, which assessed the likelihood of having a good EULAR response to TNF blockade (n = 274), compared to a poor EULAR response (n = 195), among carriers of allele G at the *PTPRC* locus (odds ratio [OR] 0.62, 95% CI 0.40, 0.95 versus carriers of allele A; *P* = 0.03) (Table 2). The genotyping success rate for rs10919563 was 98%. SNP rs11594656 at the *IL2RA* locus was also associated with a good EULAR response (OR 1.47, 95% CI 1.06, 2.04 versus no response; *P* = 0.02) but failed to demonstrate any significant association with the 6-month change in DAS28 (sample regression coefficient −0.07, 95% CI −0.21, 0.07; *P* = 0.31). All other analyses of the remaining markers revealed no compelling evidence for an association with treatment response (details available from the corresponding author upon request).

**Table 2:** Association of the rs10919563 single-nucleotide polymorphism of *PTPRC* (major allele G on chromosome 1q31.3 at position 198,700,442 bp) with the response to treatment with anti–tumor necrosis factor agents*

					Absolute change in DAS28		EULAR good response vs. no response	
						
Genotype†	Count	MAF	Baseline DAS28, mean ± SD	Change in DAS28, mean ± SD	Coef. (95% CI)	*P*	OR (95% CI)	*P*
1/1	16		6.66 ± 0.95	−2.53 ± 1.51				
1/2	224	0.12	6.73 ± 0.99	−2.32 ± 1.46	0.19 (0.09, 0.37)	0.04	0.62 (0.40, 0.95)	0.03
2/2	806		6.75 ± 1.00	−2.35 ± 1.43				

* Response to treatment was assessed as the absolute change in the Disease Activity Score in 28 joints (DAS28) over 6 months of followup, and as a good response versus no response according to the European League Against Rheumatism (EULAR) response criteria. In association analyses using the absolute change in DAS28 as the outcome measure, values are expressed as the regression coefficient (Coef.) with 95% confidence interval (95% CI), while in analyses using the EULAR response criteria as the outcome measure, values are the odds ratio (OR) with 95% CI. The multivariate model was adjusted for sex, concurrent treatment with methotrexate, DAS28 score at baseline, and Health Assessment Questionnaire score at baseline. MAF = minor allele frequency.

† 1 = major allele; 2 = minor allele.

A previous study by Cui et al demonstrated an OR of 0.55 for an association of anti-TNF treatment response with rs10919563, and the 95% confidence intervals (calculated from the genotype counts provided in that study) overlapped with the point estimate detected in the current study (95% CI 0.43, 0.77) (22). Meta-analyses of rs10919563 were therefore performed, in order to incorporate previously published data on individuals of European ancestry (22). In the fixed-effects model, rs10919563 was significantly associated with treatment response (univariate OR 0.60, 95% CI 0.47, 0.72; *P* = 5.13 × 10^−5^), thus further increasing the evidence base for this marker as a predictor of TNF blockade efficacy. These analyses revealed no evidence of genetic heterogeneity between the previous study and the current study (*P* = 0.64).

Taking into account the clinical and demographic factors predictive of TNF treatment response (DAS28 score at baseline, HAQ score at baseline, sex, and use of concurrent DMARD therapy) (Table 1), the variance in the absolute change in DAS28 at 6 months of followup was 13.5%. However, when the *PTPRC* genetic marker was included in the model, the explained variance in this outcome measure increased to 14% (results not shown).

The association of the *PTPRC* marker diminished when the stratum of anti-CCP–positive patients was investigated separately for either the absolute change in DAS28 as the outcome (sample regression coefficient 0.11, 95% CI −0.15, 0.36; *P* = 0.41) or the EULAR response (OR 0.62, 95% CI 0.39, 1.24; *P* = 0.22). Although the effect estimates were qualitatively similar to those obtained in the unstratified analyses, they were no longer significant at the 5% threshold. It should be noted that the anti-CCP status was only available for 595 patients (552 were seropositive), thus substantially reducing the power of the data in the stratified analyses.

The *PTPRC* SNP rs10919563 was investigated for drug type–specific effects, by fitting an interactive term in the analysis of absolute change in DAS28. No statistical correlation between the type of therapy used and SNP marker was observed (*P* = 0.42).

## DISCUSSION

In a large cohort of RA patients from the UK, we have corroborated the association of the *PTPRC* locus with response to anti-TNF therapy, which was first described by Cui et al in populations of Northern European descent (22). Thus, the current findings provide further support that this locus is an important marker of response to TNF blockade.

The current study investigated 29 SNP markers for correlation with treatment response, but no correction for multiple testing was applied, so as not to preclude the identification of small effects. As a result, the association at the *PTPRC* locus with treatment response could be a false positive. However, this is unlikely, because the effect was observed to be in the same direction and at a similar magnitude as that reported previously (22). Furthermore, a meta-analysis of our current data set and previous data from Cui et al (22) strengthened the evidence for association (*P* = 5.13 × 10^−5^).

PTPRC, also known as the CD45 antigen, is a transmembrane receptor–like molecule expressed on a number of immunorelevant cells. This protein plays a pivotal role in TNF signaling by being a critical regulator of signaling thresholds in immune-related cells (28). The SNP rs10919563 maps to an intron within the *PTPRC* gene on chromosome 1q31.3 (21). Examples in the literature have suggested that intronic SNPs may have a functional role by influencing levels of gene expression (29). However, the potential role of this SNP has not yet been functionally investigated.

None of the other RA susceptibility markers tested showed a correlation with treatment response, as measured both by change in the DAS28 and by the EULAR response criteria, in the current study. However, the *IL2RA* marker rs11594656 was associated with the EULAR response alone, and therefore remains of interest, warranting further investigation. Issues of power may have limited the ability to detect effects, given the modest risk conferred by alleles in studies of the anti-TNF response to date. International collaboration may be required to generate the large sample sizes required to identify such modest effects. It is interesting to note that several genetic loci reported to be associated with RA (e.g., *CD40*, *TRAF6*, and *REL*) appear to lie on the TNF signaling pathway. More powerful studies in the future may permit the investigation of possible statistical interactions between related loci. It should be noted that this is an observational study of RA, as opposed to a randomized controlled clinical trial, and this may have an important impact on the interpretation of such results.

One reason that the effect sizes observed were modest may be because the outcome tested is a relatively subjective measure of treatment success, the DAS28 score. Although a powerful means of measuring treatment response, the DAS28 is a composite score that relies on information about the swollen and tender joint counts, patient-reported general health status, and the erythrocyte sedimentation rate. Strong associations with the DAS28 have been reported in pharmacogenetic studies, in experimental conditions where there is usually a well-defined phenotype, such as a rare adverse event or correlation with an objective, biologic marker. It may be that testing a more objective measure of outcome, such as a biologic marker of inflammation, will allow the identification of genetic markers with greater effects on treatment response. This will be a focus for future work.

To date, the contribution of genetic factors in explaining treatment response to anti-TNF agents is not known. Clinical markers (e.g., baseline HAQ score, sex, anti-CCP positivity, among others) may account for up to 20% of the variance in treatment response (23), leaving a substantial proportion unexplained. The variation explained by the *PTPRC* variant is 0.5%, which alone will not be clinically useful. This SNP may provide clinical utility in the future when used as part of a treatment algorithm in combination with other genetic and clinical predictors. A number of other influences, such as patient demographics and psychological factors, may also be important contributors and require further investigation, as the mechanisms of the anti-TNF response are likely to be multifactorial.

In summary, we have performed an association study of established RA susceptibility markers with response to anti-TNF treatment in a large panel of RA patients from the UK. We have replicated the association between rs10919563 (*PTPRC)* and treatment response. These results suggest that further investigations, including fine-mapping and functional studies, at the *PTPRC* locus are now warranted.

## APPENDIX A: MEMBERS OF THE BIOLOGICS IN RHEUMATOID ARTHRITIS GENETICS AND GENOMICS STUDY SYNDICATE

Members of the UK Biologics in Rheumatoid Arthritis Genetics and Genomics Study Syndicate are as follows: A. J. Crisp, J. S. H. Gaston, F. C. Hall, B. L. Hazleman, J. R. Jenner, A. Ostor, B. Silverman, and C. Speed (Cambridge University Hospitals); D. Armstrong, A. J. Chuck, and S. Hailwood (County Durham and Darlington Acute Hospitals); L. J. Badcock, C. M. Deighton, S. C. O'Reilly, M. R. Regan, M. Snaith, G. D. Summers, and R. A. Williams (Derby Hospitals); J. R. Lambert (Doncaster and Bassetlaw Hospitals); J. Hamilton, C. R. Heycock, C. A. Kelly, and V. Saravanan (Gateshead Health); D. H. Rees and R. B. Williams (Hereford Hospitals); S. V. Chalam, D. Mulherin, T. Price, and T. Sheeran (Mid Staffordshire General Hospitals); M. Bukhari, W. N. Dodds, and J. P. Halsey (Morecambe Bay Hospitals); K. Gaffney, A. J. Macgregor, T. Marshall, P. Merry, and D. G. I. Scott (Norfolk & Norwich University Hospital); B. Harrison, M. Pattrick, and H. N. Snowden (Pennine Acute Hospitals); N. J. Sheehan and N. E. Williams (Peterborough and Stamford Hospitals); R. G. Hull, J. M. Ledingham, F. McCrae, M. R. Shaban, and A. L. Thomas (Portsmouth Hospitals); C. D. Buckley, D. C. Carruthers, R. Elamanchi, P. C. Gordon, K. A. Grindulis, F. Khattak, K. Raza, and D. Situnayake (Sandwell and West Birmingham Hospitals); M. Akil, R. S. Tattersall, D. E. Bax, S. Till, G. Wilson, J. Bouton, R. F. Kilding, L. Dunkley, and T. Tait (Sheffield Teaching Hospitals); F. Clarke, J. N. Fordham, M. J. Plant, S. Tuck (South Tees Hospitals); V. E. Abernethy, J. K. Dawson, and M. Lynch (St Helens and Knowsley Hospitals); S. Bingham, M. Buch, P. Emery, and A. Morgan (Leeds Teaching Hospitals); F. Birrell, P. Crook, H. E. Foster, B. Griffiths, I. D. Griffiths, M. L. Grove, J. D. Isaacs, L. Kay, A. Myers, P. N. Platt, D. J. Walker, P. Peterson, and W. F. Ng (Newcastle upon Tyne Hospitals); S. Bowman, C. D. Buckley, P. Jobanputra, R. W. Jubb, and E. C. Rankin (University Hospital Birmingham); E. H. Carpenter, P. T. Dawes, A. Hassell, E. M. Hay, S. Kamath, J. Packham, and M. F. Shadforth (University Hospital of North Staffordshire); S. P. Donnelly, D. Doyle, A. Hakim, and J. G. Lanham (Whipps Cross University Hospital).
